# High serum CCL20 is associated with tumor progression in penile cancer

**DOI:** 10.7150/jca.48939

**Published:** 2020-09-30

**Authors:** Miao Mo, Shiyu Tong, Wei Huang, Yi Cai, Xiongbing Zu, Xiheng Hu

**Affiliations:** 1 Department of Urology, Xiangya Hospital, Central South University, Changsha, Hunan 410008, P.R. China.; 2 Xiangya School of Medicine, Central South University, Changsha, Hunan 410008, P.R. China.

**Keywords:** penile cancer, CCL20, cancer biomarker, prognosis

## Abstract

Serum cancer biomarker has been proven to be very valuable in cancer diagnosis, disease monitoring and prognosis assessment, despite there is still a lack of serum biomarker for penile cancer (PC). Our initial analysis on public GEO dataset identified CCL20 as a top C-C motif ligand (CCL) gene enriched in PC. The patients with PC exhibited markedly higher preoperative serum CCL20 level than healthy control. The area under the curve (AUC) was 0.855 with the sensitivity of 72.4%, and specificity of 93.5% to distinguish PC. Preoperative serum CCL20 level was significantly associated with clinicopathological characteristics including T stage (*P*=0.005), nodal status (*P*=0.008), and pelvic lymph node metastasis (*P*=0.007). PC Patients with high serum CCL20 level had shorter disease-free survival compared to those with low level (*P*<0.001). Cox regression analysis showed that serum CCL20 level could serve as an independent prognostic factor for disease-free survival with a HR of 3.980 (95% CI: 1.209-13.098, *P*=0.023). Furthermore, CCL20 expression was observed in PC tissues and cell lines. Knockdown of CCL20 expression markedly suppressed malignant phenotypes (cell proliferation, clonogenesis, apoptosis escape, migration and invasion), attenuated STAT3 and AKT signaling and reduced MMP2/9 secretion in PC cell lines. Consistently, CCL20 and its receptor CCR6 exhibited correlated expression pattern in PC tissues. In conclusion, serum CCL20 level might serve as a potential diagnostic and prognostic cancer biomarker for PC. CCL20 might activate multiple downstream oncogenic signaling pathways (STAT3, AKT, MMP2/9) to promote malignant progression of PC, which may warrant further investigation in the future.

## Introduction

Although penile cancer (PC) is a rare cancer in North America and Western Europe, its incidence is much higher in some regions of South America, Asia, and Africa 1. Despite recent progress in multimodal therapies, the clinical outcome of PC still remains unsatisfactory, as the survival of PC patient has not improved during the last two decades 2. Serum-based tests have already been developed for cancer, which has been proven to be a promising noninvasive tool that aids in cancer diagnosis, disease monitoring and prognosis assessment. Serum cancer biomarkers including CEA, AFP, CA125, CA19-9 and CA15-3 have been very useful to assist cancer diagnosis and disease monitoring in various cancers 3-5. However, these serum cancer biomarkers are not very useful in PC. Although Squamous Cell Carcinoma antigen (SCCa) levels were found to be correlated with tumor burden of PC, it could not predict the clinical outcome 6. Recently, serum LAMC2 was shown to play a critical role for aggressive tumor behavior and might predict poor survival in PC 7.

Chronic inflammation is deeply involved in the development of cancers, with pro-inflammatory chemokines signaling within the tumor microenvironment contributing to tumor progression and metastasis 8. The chemokine family consists of approximately 50 small (8-14 kDa), basic proteins that are secreted by a wide range of normal and malignant cells. Chemokine C-C motif ligands (CCLs), also known as monocyte chemotactic proteins, are important chemokines that recruits monocytes, memory T cells, and dendritic cells to sites of tissue injury and infection 8. Until now, more than twenty CCLs in mammalian cells have been identified (CCL1, CCL2, CCL3, CCL5, CCL7, CCL8, CCL13, CCL15, CCL16, CCL17, CCL18, CCL19, CCL20, etc.) 9. The CCLs and its receptor Chemokine C-C motif ligand receptors (CCRs) signaling pathway participates in the physiological process including inflammation, homing of lymphocytes as well as angiogenesis 10.

In addition to their physiological roles, CCLs have also been implicated in cancer development. Aberrant expression of CCLs has been documented in many cancers. CCL2 is overexpressed in liver, breast, gastric, and thyroid cancer 11; CCL5 is overexpressed in gastric, pancreatic, breast, colorectal, prostate, liver, and thyroid cancer 12; CCL7 is markedly upregulated in lung, gastric, colorectal, and uroepithelial cancer 13. Recently, increased CCL19 expression is associated with malignant progression of prostate, colorectal, lung, and cervical cancer 14, 15. However, there is a paucity of information on the expression and clinical significance of CCLs in PC. Herein, we aimed to examine the expression of CCLs in PC tissues, and evaluate the usefulness of serum CCLs as a potential cancer biomarker for PC.

## Material and Methods

### Patient characteristics

The patients (n=76) included in this retrospective study were performed surgery and diagnosed with PC (2016-2018) at Xiangya Hospital, Central South University. Patients with prior chemotherapy or brachytherapy history were not qualified for the study. Blood samples were collected 1 day prior to surgery (preoperative) or at day 28 after surgery (postoperative). Serum samples were separated and stored at -80 ºC for later analysis. Serum samples of healthy male donors (n=46) were obtained from Health Examination Center (Xiangya hospital, Central South University) with informed consent. This study was approved by the Research Ethics Committee of Xiangya Hospital Central South University (Rev.No. 201805847). Written informed consent was obtained from the patients and participants. The clinical parameters of PC patients included age, stage, nodal status/distant metastasis, histological subtype, pathological grade, and body mass index (BMI) as well as phimosis, etc. TNM staging was assigned with reference to the American Join Committee on Cancer, 8th edition 16. All patients were prescribed a follow-up regimen based on the National Comprehensive Cancer Network guidelines, with physical examination every 3-6 months depending on nodal stage 17.

### Reagents and cell lines

Primary antibodies against CCL20 and β-actin were obtained from Abcam (Cambridge, MA, USA); Human penile cancer cell lines Penl1, Penl2, 149RCa and LM156 were kindly provided by Prof. Hui Han (Department of Urology, Cancer hospital, Sun Yat-Sen University) 18. Human foreskin fibroblasts (FSF) were obtained from the Type Culture Collection of the Chinese Academy of Sciences, Shanghai, China. These cell lines was routinely grown in Dulbecco's modified Eagle's medium supplemented with 10% fetal bovine serum. Lentiviral plasmids expressing scramble or shCCL20 was purchased from Genecopoeia Inc. (Rockville, MD). The packaging procedure for lentiviral shRNAs was conducted as we described previously 19, 20.

### Real-Time PCR

Fresh PC and matched adjacent penile tissues (AT) (n=23) were obtained from Department of Urology, Xiangya Hospital Central South University with informed consent. The mRNA levels of CCL20 in PC and AT were analyzed using real-time PCR as we described previously 19. Briefly, total RNA was extracted from tissues using TRIzol reagent and further purified using the QIAGEN RNeasy kit (Germantown, MD, USA). Total RNA (1µg) was used to generate cDNA, which was then used for the quantitative PCR using Invitrogen SYBRGreen PCR assays (Carlsbad, CA, USA). Relative gene expression was determined based on the threshold cycles (Ct values) of the CCL20 and of the internal reference gene β-Actin. PCR Primers for CCL20 and β-Actin are listed as follows:

- CCL20: Forward: 5' TCAGAAGCAGCAAGCAAC 3'; Reverse: 5' CCATTCCAGAAAAGCCACAG 3'.
- β-Actin: Forward: 5' CATGTACGTTGCTATCCAGGC 3'; Reverse: 5' CTCCTTAATGTCACGCACGAT 3'.

The relative gene expression (RE) was calculated as the fold change relative to internal reference, which was based on the following equation:


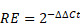
;


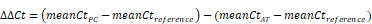


### Enzyme-linked immunosorbent assay (ELISA)

The serum CCL20 levels of the aforementioned PC patients (n=76) and healthy male subjects (n=46) were measured using Abcam CCL20 ELISA kit (Cambridge, MA, USA) according to the manufacturer's manual. The secretions of CCL20, MMP2 and MMP9 into the media collected from cell cultures were also determined by ELISA.

### Western blotting

Whole cell lysates were prepared with radio immunoprecipitation assay (RIPA) lysis buffer. The experimental procedure of Western blotting was conducted as we described previously 21. Protein blots were visualized using the Abcam enhanced chemiluminescence system (Cambridge, MA, USA).

### Cell growth analysis

Cell growth was measured by CCK-8 assay as described previously 22. The CCK-8 absorbance (OD_450_) was measured with a MK3 microplate reader (Thermo Scientific, USA).

### Caspase-3 activity assay

Cell apoptosis was determined by Caspase-3 Colorimetric Assay 22. Briefly, PC cells (5×10^5^ cells) were lysed and centrifuged, followed by enzyme reactions with chromogen. The absorbance was measured at 405 nm wavelength.

### Clonogenesis assay

Clonogenesis assay was conducted to measure the clonogenic potential of PC cells as we described previously 23. Briefly, PC cells were seeded in 6 cm culture dishes, and grown for 12 days. The number of colonies (contains >50 cells) was counted.

### Wound healing assay

Cell migration ability was measured by wound healing assay as we described previously 19. Briefly, after PC cells were grown fully confluent, a uniform scratch was made for each experiment group. The distance between the wound sides was measured immediately after the scratch or after 24 hours.

### Transwell invasion assay

Cell invasion ability was measured by transwell chamber as we described previously 20. The invaded cells on the bottom surface of the 8 µm pore membrane were stained by 0.2% crystal violet. The stained cells were eluted by acetic acid and measured with a MK3 microplate reader (Thermo Scientific, USA) at 570 nm.

### Immunohistochemistry (IHC)

Archival paraffin-embedded normal penile tissues (n=36) and PC tissues (n=40) were collected for IHC staining. These patients were performed surgery and diagnosed with PC (2017-2018) in Xiangya hospital, Central South University. IHC for CCL20 were conducted as we described previously 19. Antigen-antibody reactions (CCL20 antibody dilution, 1:200) were visualized by exposure to 3,3-diaminobenzidine and hydrogen peroxide chromogen substrate (DAKO, Denmark). The expression of CCL20 in PC cases was classified as high expression (≥ 30% of PC cells showed immunopositivity) or low expression (< 30% of PC cells showed immunopositivity).

### GEO dataset analysis

GEO dataset GSE57955 could be downloaded from NCBI GEO website (https://www.ncbi.nlm.nih.gov/geo/query/acc.cgi?acc=GSE57955). Gene expression data were processed and analyzed as described previously 24. Genes with a mean log_2_ signal ratio (penile cancer/normal glans pool) of ≥ 1.0 and ≤ -1.0 within a 99% confidence interval were considered differentially expressed.

### Statistical analysis

Statistical analyses were carried out using SPSS 16.0 software. The levels of serum CCL20 of two groups were compared by Mann-Whitney tests. The levels of serum CCL20 in three or more groups were compared by Kruskal-Wallis test followed by a Dunn's post-test. The pre- and post-operative serum CCL20 levels were compared by Wilcoxon matched pairs test. The optimal cut-off value of serum CCL20 level was determined based on receiver-operating characteristic (ROC) analysis. Kaplan-Meier curves of cancer specific survival were plotted and survival in the groups was compared by log-rank test. The prognostic factors that influence cancer specific survival were identified by univariable and multivariable Cox regression analysis. *P*<0.05 was considered statistically significant.

## Results

### CCL20 was highly expressed in PC tissues

**Figure 1:**
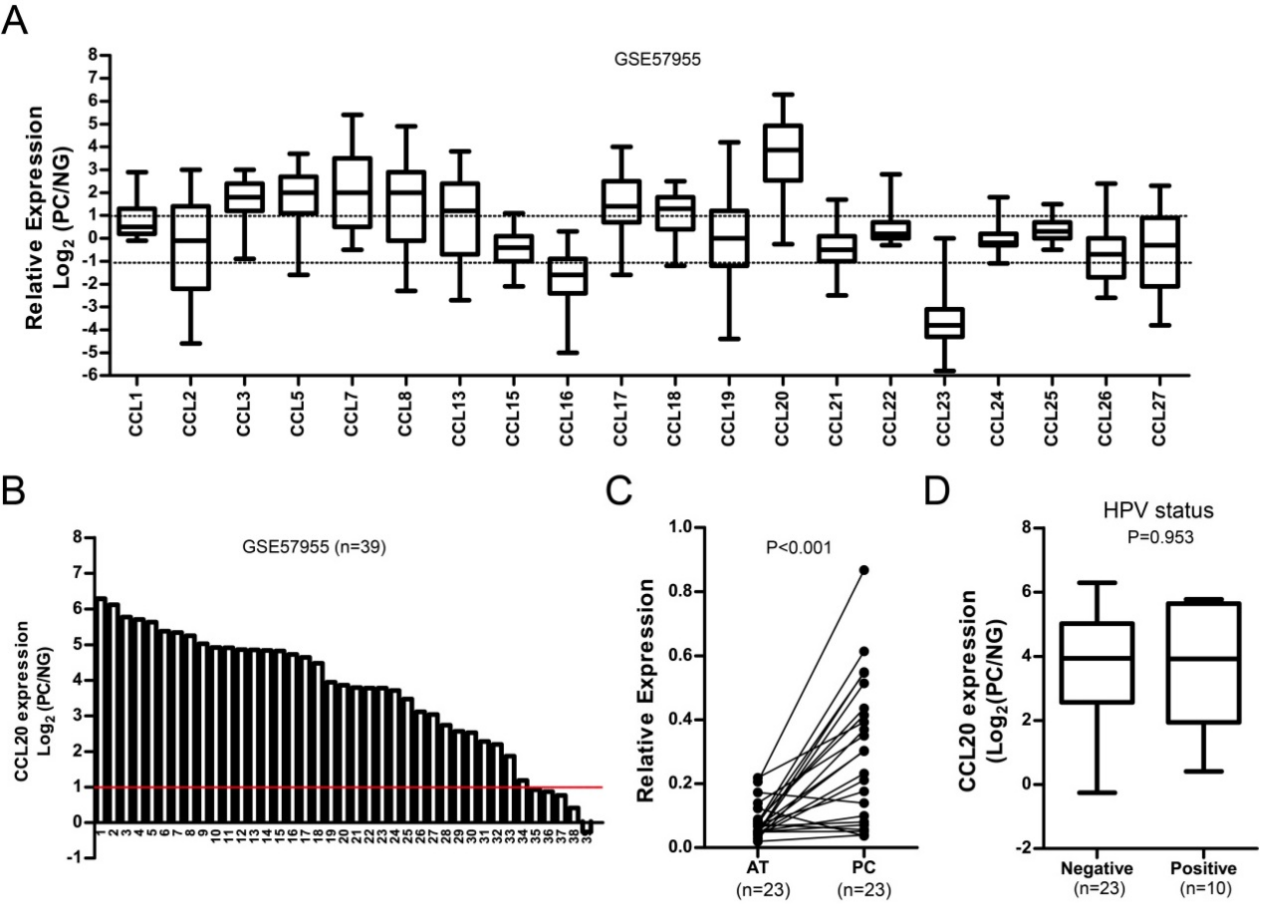
** A.** Relative mRNA expression of CCLs in PC with reference to normal glans pool (NG) in GSE57955 dataset. **B**. Waterfall plot of CCL20 expression in PC with reference to normal glans pool (NG) in GSE57955 dataset (n=39). **C**. Real-time PCR analysis on CCL20 expression in PC and matched adjacent tissues (AT) (n=23). **D**. The association between CCL20 expression and HPV status in GSE57955 dataset.

The mRNA expression of CCLs in PC (n=39) was analyzed with reference to normal glans pool (NG) in public GEO dataset GSE57955. As shown in Fig. 1A, CCL3, CCL5, CCL7, CCL8, CCL13, CCL17, CCL18, and CCL20 were highly expressed in PC (Mean Log_2_(PC/NG) ≥ 1); while CCL16 and CCL23 were low in PC (Mean Log_2_(PC/NG) ≤ 1). CCL20 was the top CCL highly enriched in PC (Mean Log_2_(PC/NG) = 3.70). More than 87.2% of PC cases (34/39) exhibited high level of CCL20 expression with reference to normal glans pool (Log_2_ (PC/NG) ≥ 1), Fig. 1B). We also examined the mRNA level of CCL20 in 23 pairs of fresh PC tissues and adjacent non-tumor tissues (AT) using real-time PCR. The result showed that the mRNA levels of CCL20 were considerably higher in PC tissues than in matched adjacent non-tumor tissues (P<0.001, Fig. 1C). We did not observe the association between human papilloma virus (HPV) infection and CCL20 expression in GSE57955 dataset, as CCL20 expression in HPV^-^ cases did not significantly differ from those HPV^+^ cases (Fig. 1D, *P*=0.953).

### Preoperative serum CCL20 level was significantly elevated in PC patients

**Figure 2:**
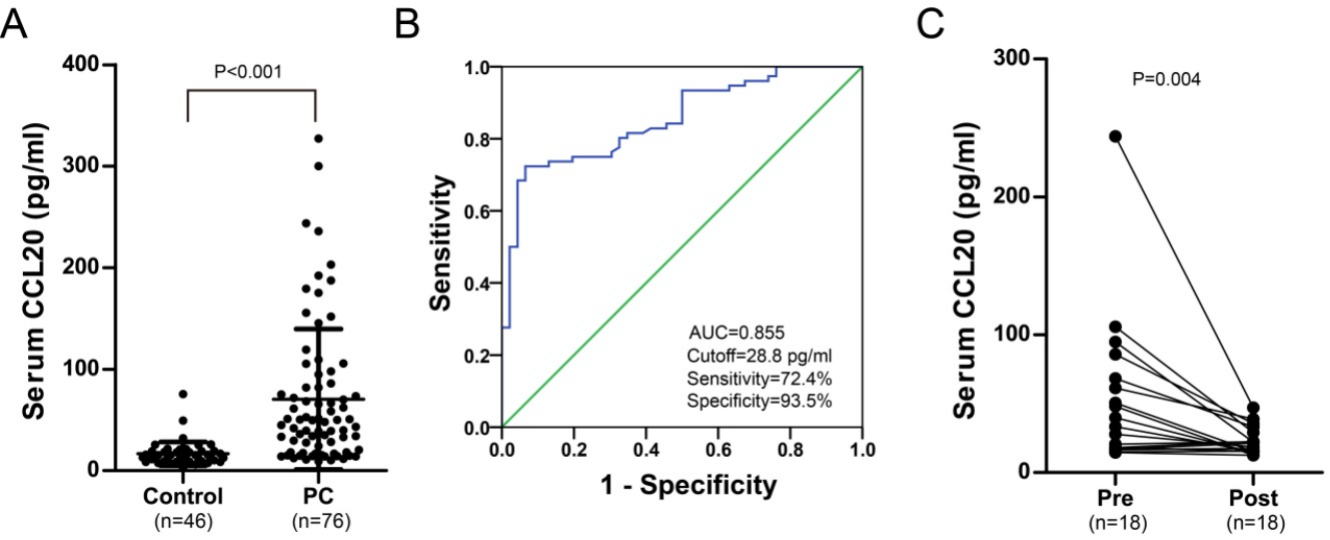
**A**. Serum CCL20 level in preoperative PC cohort (n=76) and healthy male control (n=46). **B**. ROC curve analysis of the diagnostic value of serum CCL20 level in PC patients. **C**. Serum CCL20 level in the matched preoperative/postoperative PC cohort (n=18).

A total of 76 men diagnosed with PC were enrolled in this study. Detailed summary of patient and tumor characteristics including treatment plan, TNM stage, histological subtype and pathological grade was shown in Table 1. Serum CCL20 levels were measured in healthy control (male) and PC patients. Preoperative serum CCL20 level was significantly higher in PC cohort (70.4 ± 69.3 pg/ml) than those from healthy male subjects (16.6 ± 11.8 pg/ml) (*P* < 0.001, Fig. 2A). The area under the curve (AUC) was 0.855 with the sensitivity of 72.4%, and specificity of 93.5% to distinguish penile cancer (Cutoff =28.8 pg/ml, Fig. 2B). Moreover, Serum CCL20 levels were significantly decreased after PC surgery (*P* = 0.004, Fig. 2C).

### Preoperative serum CCL20 level was associated with tumor progression and unfavorable clinical outcome

**Figure 3:**
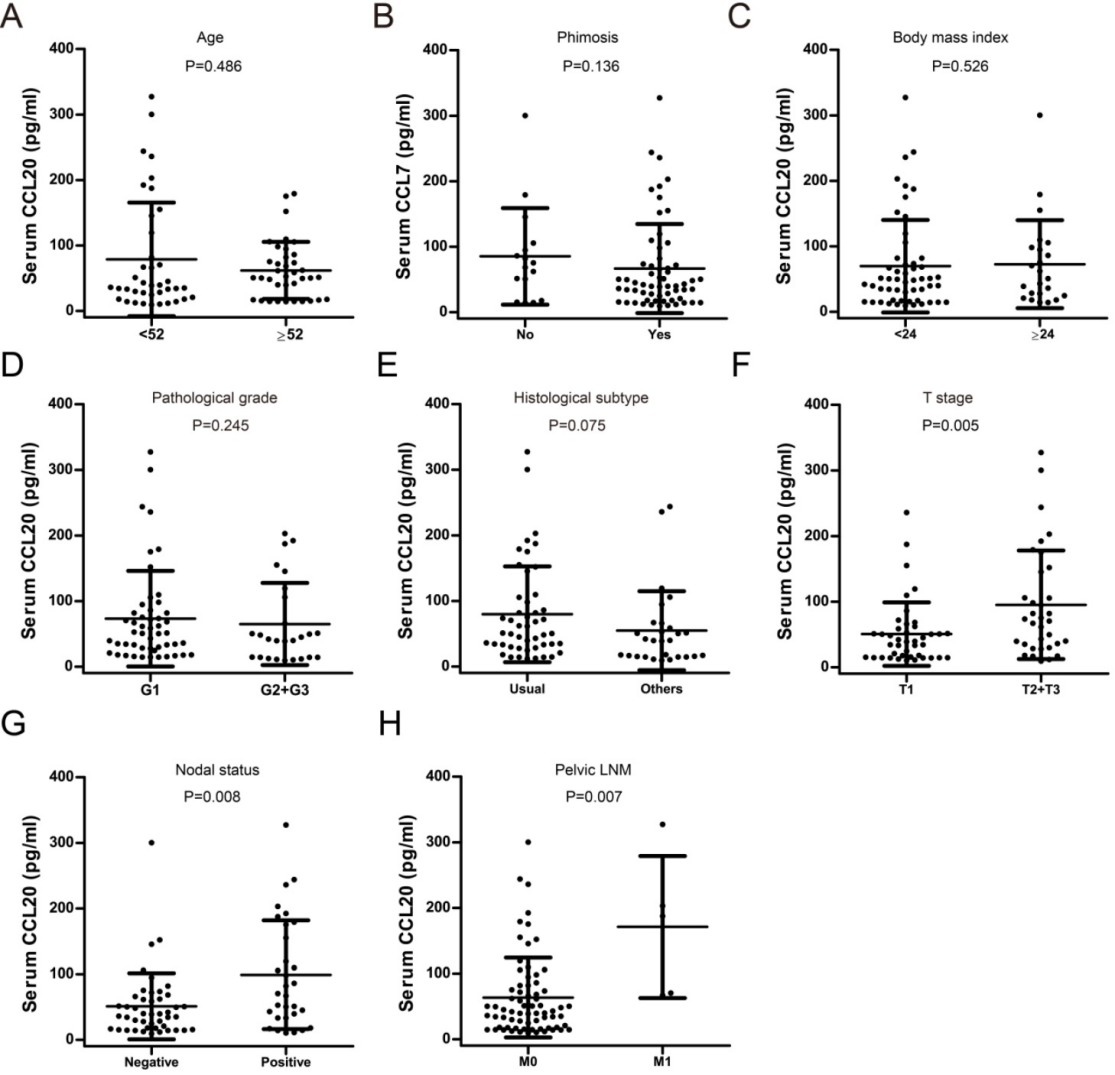
Association between preoperative serum CCL20 level and clinical parameters in our PC cohort. **A**. Age; **B**. Phimosis; **C**. BMI index; **D**. Pathological grade; **E** Histological subtype; **F**. T stage. **G**. Nodal status. **H**. Pelvic LNM.

**Figure 4:**
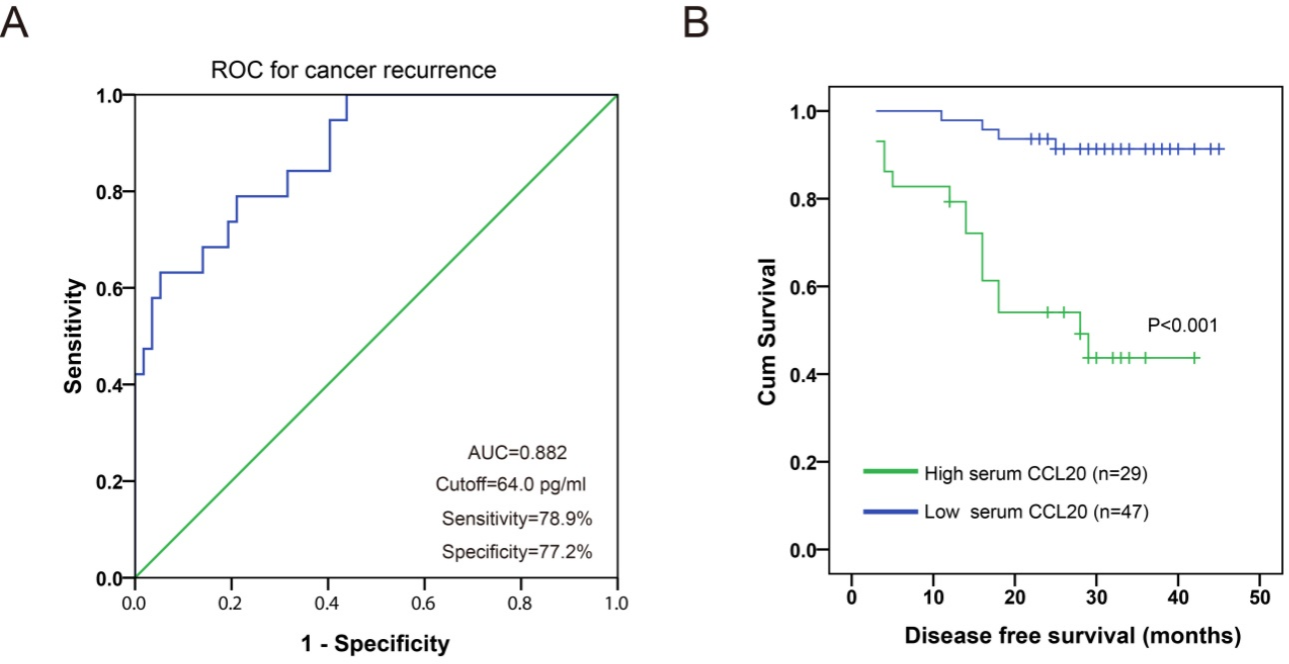
** A.** ROC curve for preoperative serum CCL20 level with reference to cancer recurrence. **B.** PC patients with high preoperative serum CCL20 level exhibited shorter disease free survival.

The association between preoperative serum CCL20 level and clinicopathological parameters (age, body mass index, pathological grade, phimosis, histological subtype, TNM stage) was analyzed. As shown in Fig. 3, preoperative serum CCL20 level was significantly associated with oncologic parameters including T stage (*P*=0.005), nodal status (*P*=0.008), and pelvic lymph node metastasis (LNM) (*P*=0.007). Preoperative serum CCL20 level was not significantly associated with body mass index (*P*=0.526), phimosis (*P*=0.136), age (*P*=0.486), histological subtype (*P*=0.075), and pathological grade (*P*=0.245). ROC analysis showed that serum CCL20 level had a sensitivity of 78.9% and a specificity of 77.2% with reference to cancer recurrence (cutoff value: 64.0 pg/ml, AUC=0.882, Fig. 4A). Survival analysis showed that PC patients with high serum CCL20 level (≥ 64.0 pg/ml) exhibited unfavorable disease free survival (*P*<0.001) (Fig. 4B).

Univariable Cox regression analysis showed that inguinal nodal status (P<0.001), pelvic LNM (P<0.001), T stage (P=0.021), and higher preoperative serum CCL20 level (P<0.001) were associated with shorter cancer specific survival in our PC cohort (Table 2). Meanwhile, multivariable Cox regression analysis indicated that nodal status (P=0.009), pelvic LNM (P=0.006), and higher preoperative serum CCL20 level (P=0.023) could serve as independent prognostic factors for disease free survival (Table 2).

### Expression of CCL20 in normal penile tissues and PC tissues

**Figure 5:**
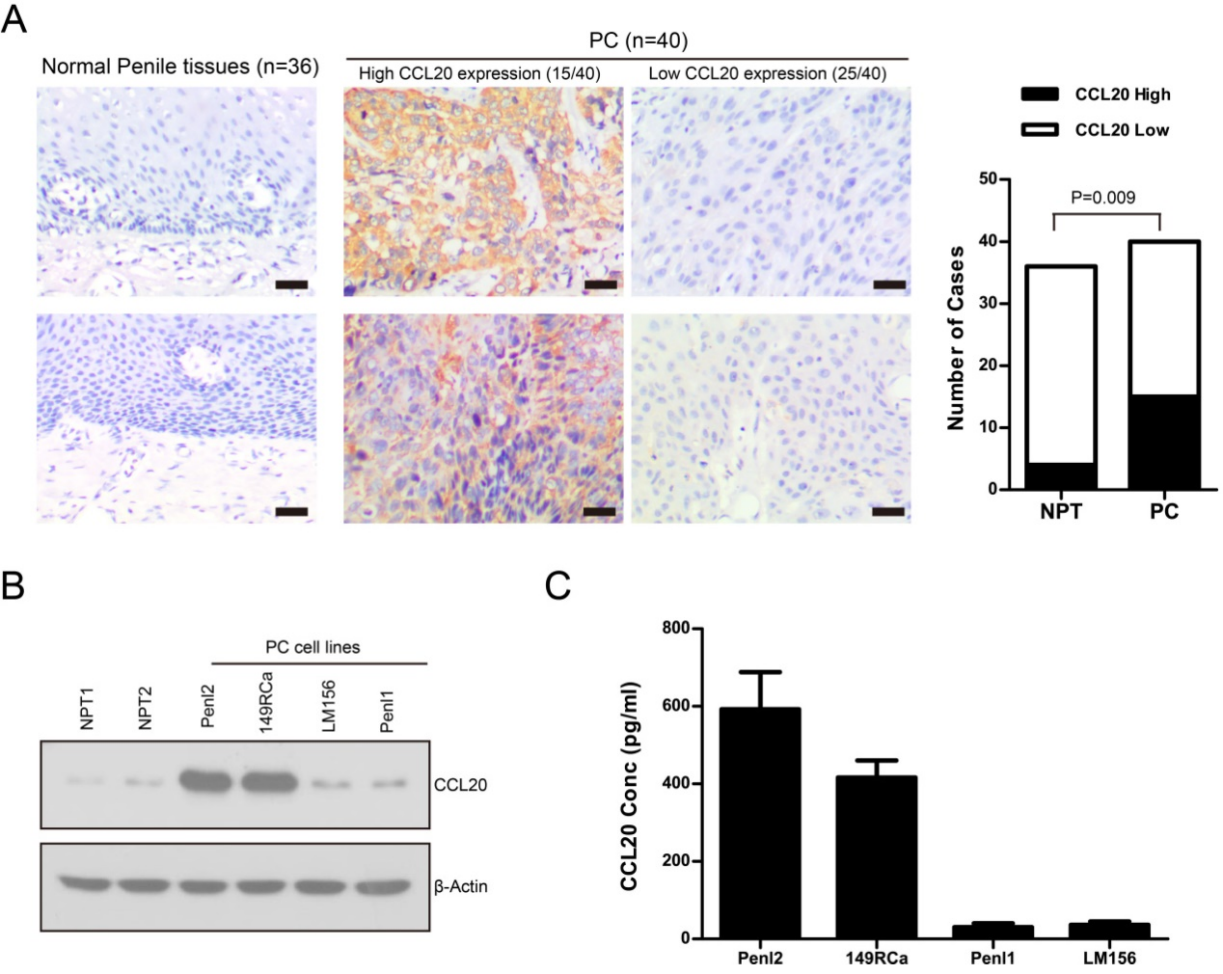
** A**. Expression of CCL20 in normal penile tissues (NPT, n=36) and PC tissues (n=40). Representing micrographs showed high or low CCL20 expression in normal penile tissues and PC, respectively. Bars: 50 µm. PC vs. NPT, P=0.009.** B.** Expression of CCL20 in normal penile tissues (NPT1, NPT2) and PC cell lines. **C**. CCL20 level in culture supernatant of PC cell lines.

CCL20 expression in normal penile tissues (NPT, n=36) and PC tissues (n=40) was analyzed by IHC. CCL20 exhibited mostly cytoplasmic staining in PC cells (Fig. 5A). Overall, 37.5% PC cases (15/40) exhibited high CCL20 expression (Fig. 5A). The expression of CXCL13 in PC (15/40) was much higher than that in normal penile tissues (4/36) (*P*=0.009; Fig. 5A). The protein expression of CCL20 was also analyzed in normal penile tissues (NPT1, NPT2) and a panel of PC cell lines (Penl1, Penl2, 149Rca and LM156). CCL20 expression was low in normal penile tissues (NPT1, NPT2); whereas high CCL20 expression was seen in PC cell line Penl2 and 149RCa (Fig. 5B). Consistently, ELISA analysis showed that high CCL20 secretion was detected in culture supernatant from PC cell line Penl2 and 149RCa (Fig. 5C).

### Knockdown of CCL20 suppressed cell proliferation, migration and invasion in penile cancer cell lines

**Figure 6:**
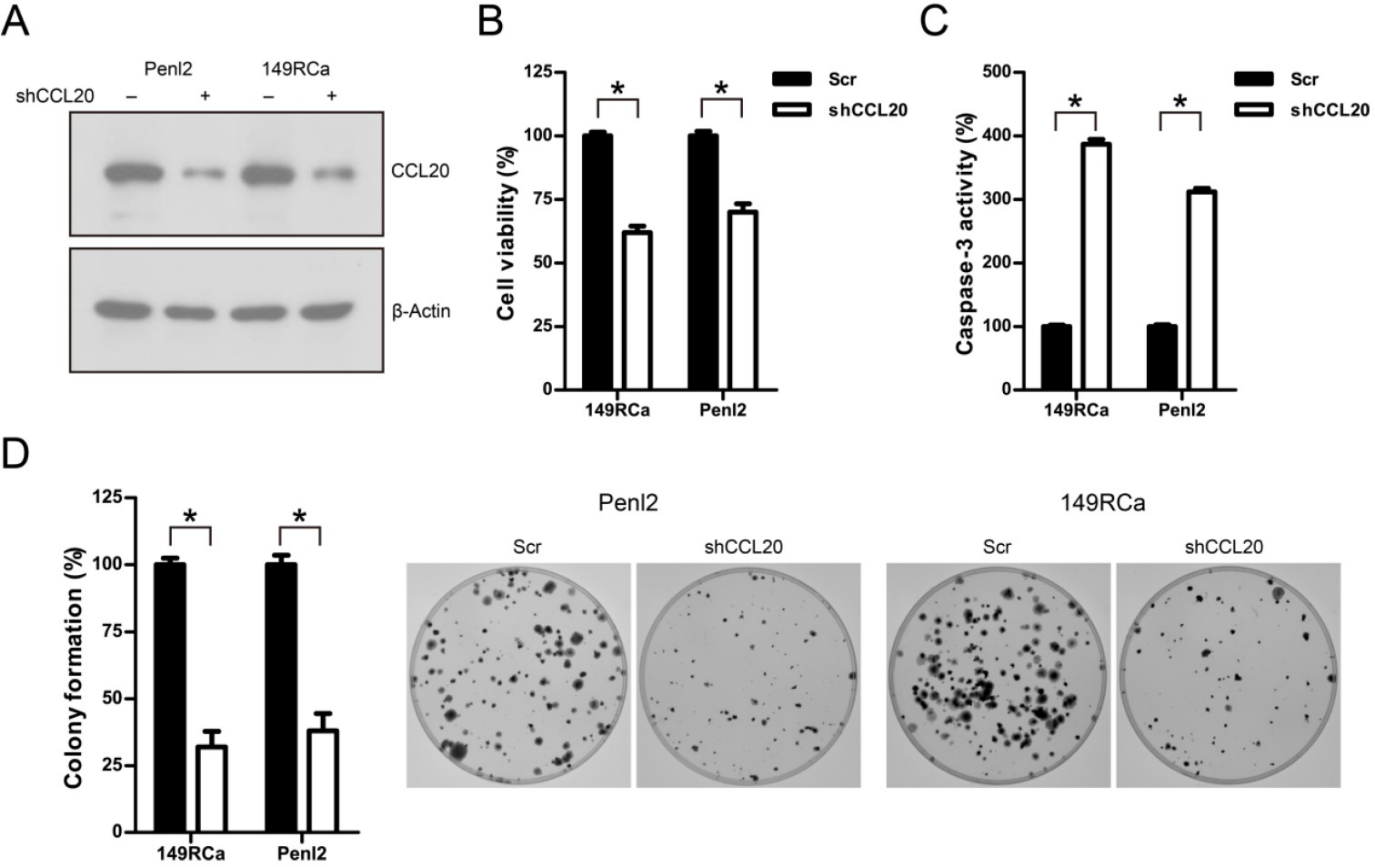
** A.** Western blotting analysis on CCL20 expression following shRNA-mediated knockdown in Penl2 and 149RCa cells. **B.** Depletion of CCL20 expression suppressed cell growth of PC cells. The cell viability in Scr control was regards as 100%. **C.** Depletion of CCL20 expression reduced clonogenesis of PC cells. The colony formed in Scr control was regards as 100%. n=3, *P<0.05. **D**. Knockdown of CCL20 induced caspase-3 activity in Penl2 and 149RCa cells. The caspase-3 activity in Scr control was regards as 100%. n=3, *P<0.05.

**Figure 7:**
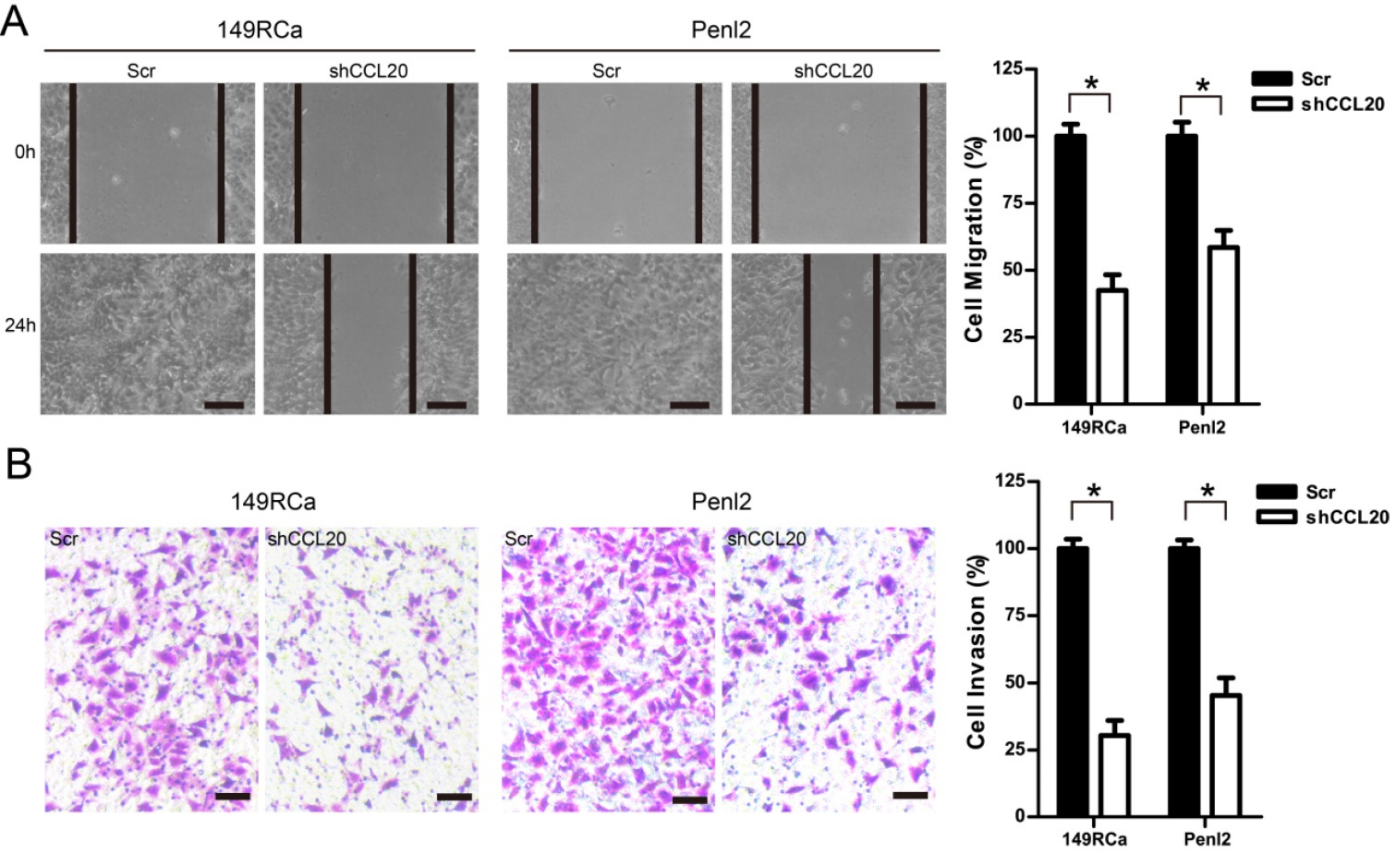
**A**. Knockdown of CCL20 expression inhibited cell migration of PC cells. Bars: 100 µm. The cell migration in Scr control was regards as 100%. n=3, *P<0.05. **B.** Knockdown of CCL20 expression inhibited transwell invasion of PC cells. Bars: 50 µm. The cell invasion in Scr control was regards as 100%. n=3, *P<0.05.

We further investigated the oncogenic function of CCL20 in PC cell line 149RCa and Penl2. CCL20 expression was remarkably decreased by shRNAs targeting CCL20 (Fig. 6A). CCK-8 assay showed that knockdown of CCL20 markedly suppressed cell proliferation compared to Scr control in 149RCa and Penl2 cells (*P*<0.05, Fig. 6B). However, caspase-3 activity was increased following CCL20 knockdown in 149RCa and Penl2 cell lines (*P*<0.05; Fig. 6C). Colony formation in shCCL20 group was greatly attenuated compared to Scr control group in 149RCa and Penl2 cells (*P*<0.05, Fig. 6D). Moreover, wound healing assay showed that cell migration was remarkably reduced in shCCL20 group compared to Scr control (*P*<0.05, Fig. 7A). Further, transwell invasion assay showed that depletion of CCL20 expression significantly inhibited the invasiveness of PC cells compared to Scr control (Fig. 7B, *P*<0.05).

### CCL20 regulated downstream STAT3 and AKT signaling pathway and matrix metalloproteinase MMP2/9 secretion

**Figure 8:**
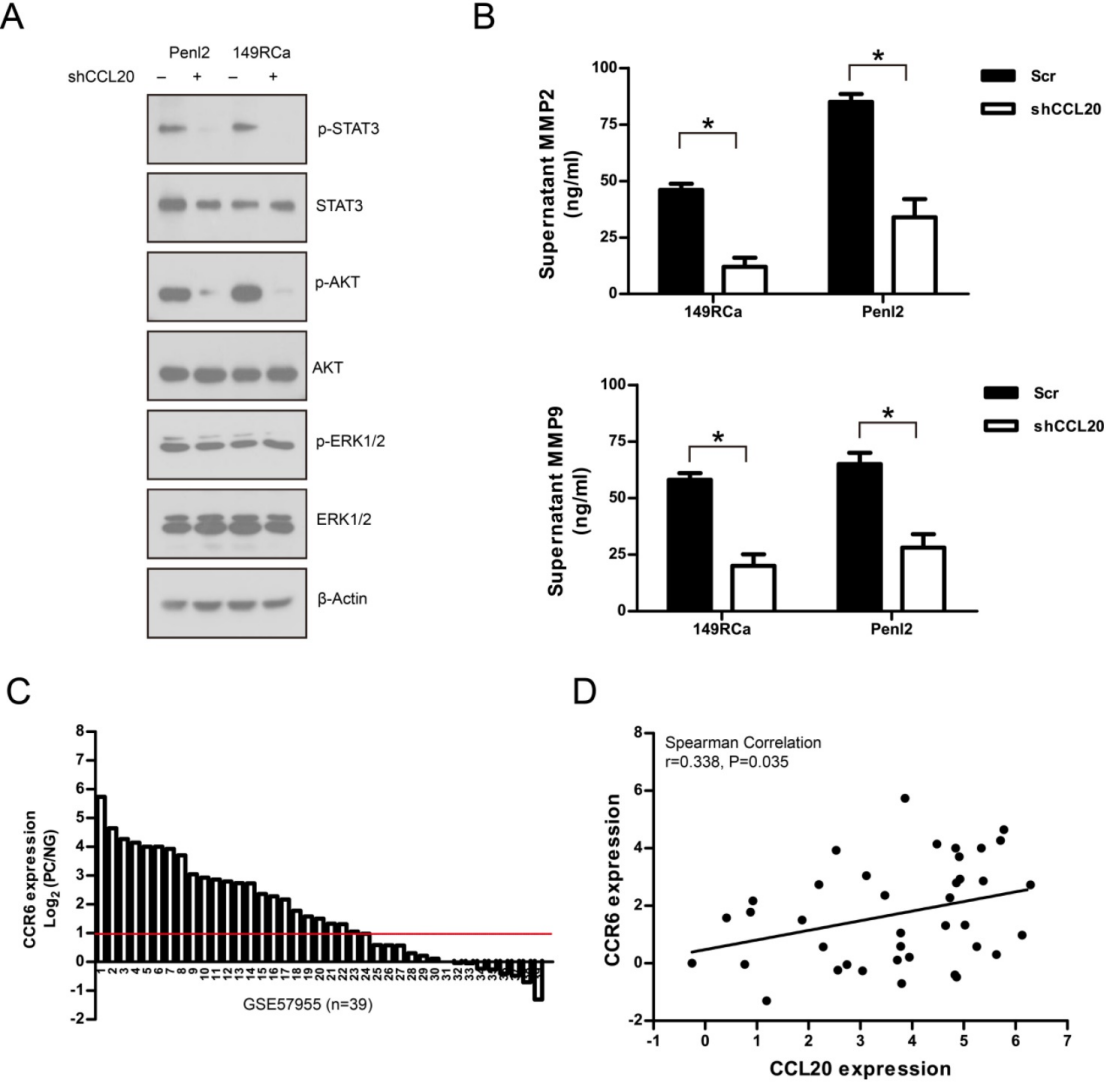
** A.** Effect of CCL20 depletion on cancer-related signaling pathways in PC cell lines. **B**. Knockdown of CCL20 reduced MMP2/9 secretion in PC cell lines. n=3, *P<0.05. **C**. Waterfall plot of CCR6 expression in PC dataset GSE57955 (n=39). **D**. Correlated expression between CCL20 and CCR6 in PC dataset GSE57955 (n=39).

The CCL20-related signaling pathways including PI3K/AKT, ERK1/2 and STAT3 were analyzed using Western blotting. As shown in Fig. 8A, knockdown of CCL20 greatly attenuated p-STAT3 and p-AKT levels without affecting p-ERK1/2 in 149RCa and Penl2 cells. Meanwhile, ELISA assay revealed that depletion of CCL20 reduced secretion of two invasion/metastasis-related molecules matrix metalloproteinase MMP2 and MMP9, as compared with Scr control (*P*<0.05) (Fig. 8B). We further evaluated the expression of CCL20 receptors CCR6 in GSE57955 dataset. As shown in Fig. 8C, CCR6 expression was high in 61.5% (24/39) PC cases (Mean Log_2_ (PC/NG) = 1.71). Correlated CCL20:CCR6 expression was observed in GSE57955 dataset (Pearson correlation r=0.338, *P*=0.035, Fig. 8D).

## Discussion

Chronic inflammation contributes to proliferation and survival of tumor cells, angiogenesis and metastasis via multiple mechanisms 25. Understanding inflammation -related mechanisms may help to identify potential target for therapeutic intervention. Chemokine CCL/CCR signaling within the tumor microenvironment of many cancers is known to enhance tumor progression via activation of signaling pathways promoting proliferation, angiogenesis, migration, invasion and cell survival 26. However, the expression patterns as well as functions of these CCLs still remain poorly understood in PC. To our knowledge, our study may be the first to analyze the expression patterns of CCLs in PC. Our analysis showed that several CCLs (CCL3, CCL5, CCL7, CCL8, CCL13, CCL17, CCL18 and CCL20) were remarkably upregulated in PC, suggesting these CCLs might play an important role in PC carcinogenesis. Further study would be necessary to investigate the possible oncogenic function of these CCLs in PC.

CCL20, also known as macrophage inflammatory protein 3 Alpha (MIP-3α), is a chemotactic factor and attractant for lymphocytes and neutrophils 27. Recently, the expression and clinical relevancy of CCL20 in human cancers has been documented by a number of studies 28-30. Serum CCL20 was identified as independent prognostic markers for colorectal cancer, which also exhibited a good performance in the diagnosis of early stage disease 31. Serum CCL20 level was also shown to be increased markedly in patients with esophageal cancer, and was associated with cancer occurrence and metastasis 32. In nasopharyngeal carcinoma, serum CCL20 level was significantly higher in untreated patients, recurrent patients and patients with distant metastases 33. Serum CCL20 may also be used for the detection of hepatocellular carcinoma in HCV-infected patients with comparable specificity and higher sensitivity than Alpha fetoprotein (AFP) 34. Herein, we identified CCL20 as top CCL highly expressed in PC in GSE57955 dataset. HPV is an important contributing factor for about 30~40% of PC 35. However, our findings did not reveal the association between CCL20 expression and HPV status, suggesting aberrant CCL20 expression in PC might not be caused by HPV infection. Some studies on other cancers suggested that CCL20 expression might be driven by cancer-related signaling including nuclear factor-κB and RANK/RANKL 36, 37. However, how CCL20 is upregulated in PC still awaits further investigation.

Consistent with our findings on tissue CCL20, we showed that PC patients exhibited markedly higher preoperative serum CCL20 level than healthy control. Serum CCL20 exhibited the sensitivity of 72.4% and specificity of 93.5% to distinguish PC, Further, we showed that preoperative serum CCL20 level was significantly associated with clinicopathological characteristics including T stage, nodal status, and pelvic LNM in PC; high preoperative serum CCL20 level could serve as an independent prognostic factor for unfavorable disease free survival in PC. Therefore, serum CCL20 might serve as a potential diagnostic and prognostic biomarker for PC. However, due to the limitation of our retrospective study (single center, small cohort, relatively short follow-up period, diversity of treatment, etc.), large-scale prospective study would be necessary to further validate the potential value of preoperative serum CCL20 as a diagnostic and prognostic biomarker for PC.

CCL20 has been recently correlated with malignant phenotypes (uncontrolled cell proliferation, invasion, metastasis, etc.) in many cancers. In endometrial cancer, CCL20 mediates RANK/RANKL-induced epithelial-mesenchymal transition (EMT) and tumor metastasis 36. In ovarian cancer, CCL20 could promote tumor progression in the peritoneal cavity 37. CCL20 could also promote cell proliferation and metastasis in laryngeal cancer 38. In this study, we showed that CCL20 expression could be detected in PC tissues, cell lines and culture supernatants, suggesting CCL20 might act in an autocrine manner in PC. Further, we showed that knockdown of CCL20 suppressed cell proliferation, apoptosis escape, clonogenesis, migration and invasion in PC cell lines, suggesting CCL20 might be crucial to regulate tumor progression in PC.

Multiple oncogenic signaling pathways including STAT3, PI3K/AKT, and metaloproteinases MMP2/9 contribute to PC carcinogenesis. Stankiewicz and Chaux et al. showed that AKT expression positively correlated with tumor grade and prognosis of PC 39, 40. Yang et al identified STAT3 as one of the top oncogenic pathways upregulated in PC 41. Campos et al indicated that high MMP-9 expression was an independent risk factor for disease recurrence in PC 42. Our present findings reveal that knockdown of CCL20 attenuated AKT and STAT3 signaling and reduced MMP2/9 secretion, suggesting the effect of CCL20 might result from its function in activating oncogenic signaling pathways (STAT3, AKT) and enhancing the expression of metastasis-related MMP2/9, thus promoting the tumor progression of PC. Nevertheless, CCL20 expression was significantly correlated with its receptor CCR6 in PC, suggesting CCL20/CCR6 signaling might play a crucial role in PC carcinogenesis. Although CCL20 is known to induce ERK1/2 signaling in lung cancer cells 43, we did not observed the attenuation of ERK1/2 signaling following knockdown of CCL20 in PC cell lines, suggesting that CCL20 signaling might activate differential downstream signaling dependent in specific cancer types.

In conclusion, we have shown that serum CCL20 is closely associated with tumor progression and might serve as diagnostic and prognostic biomarker for PC. Further, CCL20 might activate multiple downstream oncogenic signaling (STAT3, AKT, MMP2/9) to promote malignant phenotypes in PC. New discoveries in the CCL20 signaling would also aid clinical decision-making for PC patients, bringing us closer to the promise of translational precision medicine.

## Figures and Tables

**Table 1:** Clinicopathologic characteristics of PC patient cohort

Parameters	Cases (%)
**Age (year)**	
>52	37 (48.7%)
≤52	39 (51.3%)
**Body mass index (kg/m^2^)**	
<24	53 (69.7%)
≥24	23 (30.3%)
**Phimosis**	
Yes	60 (78.9%)
No	16 (21.1%)
**Penile Surgery**	
Penile preservation	19 (25.0%)
Partial penectomy	52 (68.4%)
Radical penectomy	5 (6.6%)
**Pathological Grade**	
G1	50 (65.8%)
G2	22 (28.9%)
G3	4 (5.3%)
**Histological subtype**	
Usual	48 (63.2%)
Papillary	6 (7.8%)
Warty	12 (15.8%)
Verrucous	10 (13.2%)
**T stage**	
T1	42 (55.3%)
T2	30 (39.5%)
T3	4 (5.2%)
**Nodal status**	
Negative	45 (59.2%)
Positive	31 (40.8%)
**Pelvic LNM**	
M0	71 (93.4%)
M1	5 (6.6%)
**Inguinal lymphadenectomy**	
No	46 (60.5%)
Yes	30 (39.5%)

**Table 2:** Cox univariate and multivariate proportional hazard model for factors affecting disease-free survival in PC cases

Clinical parameters	Univariate analysis	Multivariate analysis	
	*P* value	HR (95%CI)	*P* value
**T stage** (T1 vs. T2+T3)	0.021		0.656
**Pathological grade**(G1 vs. G2+G3)	0.121		
**Histological subtype**(Usual vs. Others)	0.279		
**Nodal status**(Negative vs. Positive)	<0.001	5.785 (1.553-21.543)	0.009
**Pelvic LNM**(No vs. Yes)	<0.001	4.9993 (1.581-15.765)	0.006
**High serum CCL20**(≥64.0 pg/ml vs. <64.0 pg/ml)	<0.001	3.980 (1.209-13.098)	0.023

## Abbreviations

PC: Penile cancer
CCL20: Chemokine C-C motif ligand 20
CCRs: Chemokine C-C motif ligand receptors
CEA: Carcinoembryonic antigen
AFP: Alpha-fetoprotein
SCCa: Squamous Cell Carcinoma antigen
ELISA: Enzyme-linked immunosorbent assay
IHC: Immunohistochemistry
Pelvic LNM: Pelvic lymph node metastasis
HPV: Human papilloma virus
